# Cancer Is to Embryology as Mutation Is to Genetics: Hypothesis of the Cancer as Embryological Phenomenon

**DOI:** 10.1155/2017/3578090

**Published:** 2017-05-03

**Authors:** Jaime Cofre, Eliana Abdelhay

**Affiliations:** ^1^Laboratório de Embriologia Molecular e Câncer, Universidade Federal de Santa Catarina, Sala 313b, 88040-900 Florianópolis, SC, Brazil; ^2^Divisão de Laboratórios do CEMO, Instituto Nacional do Câncer, Rio de Janeiro, RJ, Brazil

## Abstract

Despite numerous advances in cell biology, genetics, and developmental biology, cancer origin has been attributed to genetic mechanisms primarily involving mutations. Embryologists have expressed timidly cancer embryological origin with little success in leveraging the discussion that cancer could involve a set of conventional cellular processes used to build the embryo during morphogenesis. Thus, this “cancer process” allows the harmonious and coherent construction of the embryo structural base, and its implementation as the embryonic process involves joint regulation of differentiation, proliferation, cell invasion, and migration, enabling the human being recreation of every generation. On the other hand, “cancer disease” is the representation of an abnormal state of the cell that might happen in the stem cells of an adult person, in which the mechanism for joint gene regulating of differentiation, proliferation, cell invasion, and migration could be reactivated in an entirely inappropriate context.

## 1. Cancer and the Environment

The concept of environment is often used with a broad scope and includes all nongenetic factors such as diet, lifestyle, and infectious agents. At the current juncture of the XXI century, cancer disease should not be dissociated from the environment and external stimuli, which are considered as the causes of most human cancers [1, 2]. From our point of view, the environmental stimuli are responsible for reactivating intrinsic mechanisms of gene regulation norms that could trigger the “cancer process.” Then, such mechanisms may be set sequentially during the embryonic period and used in the embryo construction as well as in situations of particular tissue reconstruction of an adult person. In countercurrent with this way of thinking, it is strongly considered that mutations (at random or by chance) [3, 4] or environmental factors inducing mutations are the leading causes of cancer over, for example, environmental factors that can be activating the cell's normal regulatory mechanisms.

There are “sporadic” and hereditary cancers when they are considered from the clinical point of view. “Sporadic” cancers account for over 95% of human cancers. On the other hand, hereditary cancers (less than 5% of the total population cancers), such as adenomatous polyposis coli, although they present DNA mutations and are present in all body cells, appear only or primarily in one or a few organs [5]. Also, the epigenetics consolidated a formal theory of carcinogenesis [6] that could explain cancer predisposition in humans related to epimutations (an epigenetic hereditary abnormality in gene expression) transmitted from mother to child [7]. In any case, the contribution of inherited factors (genetic or epigenetic) for cancer development is believed to be relatively small [8].

Furthermore, the epidemiological study shows that the most common cancers in a population are relatively rare in others and with evident variations over time [9]. For example, in Brazil, it is possible to find regional differences in the federation states about the gross rates incidence per one hundred thousand women concerning breast cancer. Thus, it shows preponderance in the Southeast states. Furthermore, the uterine cervix has preponderance in the Northeastern and Northern regions of Brazil [10]. Then, these data indicate that lifestyle or environmental stimuli could be the primary causes of these types of cancers. Also, the use of statistical models for data analyses from large samples of twins (monozygotic and dizygotic) helped to estimate the magnitude of genetic and environmental effects on cancer susceptibility. They confirmed that the inherited genetic factors have a minor contribution to the susceptibility of most types of cancer, and the “environment” could have a significant role in causing Sporadic cancer [11, 12].

It is also possible to verify by the recent progress in developmental biology and cancer biology that human embryonic cells are very similar in their phenotype to the cancerous cells. Some differences between normal proliferative cells and cancer cells are addressed in DeBerardinis et al. [13]. Then, the authors will refer to the gastrulation process by the name of “cancer process” to emphasize the process naturalness and its importance in human embryogenesis. In agreement with that similarity, the genes involved in carcinogenesis (from now on referred to as “cancer disease”) are a set of genes activated simultaneously that effectively recapitulate the embryogenesis. In other words, the human embryonic genes are reexpressed in cancer cells [14–19]. Therefore, the embryonic origin discussion of “cancer disease” should have been extended, and prevention discussion should follow the same pace. It seems to be obvious, in the current context, in which there is strong scientific basis of lung cancer being associated with smoking and its reduction relates to habits changes, and also, that the hepatocellular carcinoma decreases in vaccinated populations against hepatitis B virus [9]. Surprisingly, despite the data shown before, there is no current trend in cancer research to solve first the issues inherent to the disease origin and its relation to the environmental stimuli. Then, in the present article, the authors are going to discuss their ideas about the embryological genesis of human cancer.

## 2. Cancer and Genetics

Cancer origin has focused almost exclusively on only one theory: the somatic mutation theory (SMT). It was first enunciated, in 1914, by Theodor Boveri in his book* The Origin of Malignant Tumors* [20, 21]. Although the author acknowledges that “I write about this problem as a zoologist and I have no personal experience worth mentioning in any of the numerous specialized fields of tumor research,” he established as essential principles of his proposal that “a malignant tumor cell is a cell with a specific defect; it has lost properties that a normal tissue cell retains.” The specific defect might be a permanent change or “a particular abnormal composition of the chromatin,” which in the terms described by Boveri represent “a disorder in certain chromosomes produced by a hereditary condition; destruction of chromosomes by intracellular parasites; damage of particular chromosomes by external agents that spare others…”. A significant aspect of the proposal is that the tumorigenic primary cell “that harbors a specific faulty assembly of chromosomes as a consequence of an abnormal event” could trigger on an unbridled cell proliferation “that the primordial cell passes to its progeny so long as these continue to multiply by normal mitotic binary fission.” From our point of view the so-called “particular abnormal composition of the chromatin” was enshrined, in science, by DNA discovery as genetic material [22] and its chemical structure [23] and, fundamentally, by a phrase that could change or seal the fate for the faithful and devout followers of the theory. “it has not escaped our notice that the specific pairing we have postulated immediately suggests a possible copying mechanism for the genetic material” [23]. Thus, “the lesions within the chromosomes” (which later became mutations, a word coined by Morgan [24]) that “passes to its progeny” gained credibility and consolidated as the dominant factor in the origin of cancer.

Are mutations the real mechanism that causes cancer or they appear as a result of cancer and become a significant part of spontaneous neoplasia of biology in vivo? We know that cancer is a potentiated disease by mutations in somatic cells, and these mutations are not evenly distributed in the human genome. On the contrary, they exhibit very complex “mutational landscapes” often characterized by a large number of single nucleotide substitutions (SNS), which are found throughout the genome [25–28]. The SNS patterns seem to depend on the cancer type, number of cell division leading to the tumor initiation and progression, and, also, the distinct specific patterns of chromosomal organization (epigenetic/epigenomics) in different tissues [29–31]. The epigenomics organization could be a major determinant of mutational landscape observed in cancer.

Similar to these latest observations, the embryonic development is responsible for establishing cell differentiation, in the final analysis [32]. It also seems that, in embryogenesis, fundamental epigenetic changes such as alterations in the chromatin structure and nuclear organization contribute to defining different patterns of gene expression during cell differentiation [33]. We thought that determinants of mutational landscapes could be defined in the embryogenesis. A comparison of epigenetic regulation mechanisms directly involved in gene expression between well-characterized populations of adult stem cells (ASC) and embryonic stem cells (ESC) revealed that different epigenetic signatures characterize different populations of stem cells [34]. In other words, the epigenetics/epigenomics organization should emerge during embryogenesis and should link directly to cell differentiation [35, 36]. Therefore, cell differentiation capacity is primarily ruled by the chromatin structure, which directly affects its accessibility and the repertoire of available transcription factors [37]. Thus, to describe the epigenetic signature of a determined cell type, after differentiation, is a consensus that we should consider by three aspects in an integrated way: changes in nuclear organization, replication time, and global modifications in the chromatin [35]. Accordingly, mutations distributions in the genome, in several independent samples and various types of cancer, demonstrated that chromatin accessibility, their epigenetic modifications (epigenomics organization), and replication time should explain up to 86% of the rate variance of genome mutation studied in different types of cancers [38]. Then, mutations are following a preestablished pattern by cell differentiation standard and, therefore, they make us think that such changes are, perhaps, a consequence of initial cancer development and not the other way around.

Thus, the best predictors of local somatic mutation density or cancer mutation profile are related to the epigenomics organization associated with the cell type of origin of the corresponding malignancy. Therefore, the origin cell type of cancer can be determined accurately by using mutations distribution throughout its genome as a base [38], and that observation indicates again strongly that chromosomal abnormalities or mutations often observed in cancer disease are the results of epigenomics organization. Then, a genome mutational landscape associated with a particular type of cancer includes a source of information on the identity and epigenetic organization of its origin cell and the responsible embryonic process of its genesis.

Even though the experimental results open the way for a critical discussion on embryonic development role in cancer origin, it is not what we see in practice. Justifications used by most of the authors relate to the following: (1) most somatic mutations observed in the studied cancers may appear before the epigenetic alterations associated with neoplastic progression. (2) Advanced tumors can undergo specific epigenetic changes that distinguish them from other tumors of the same type [38] or (3) more radical arguments considered that most of the analyzed mutations reside in noncoding parts of the genome; and therefore, these patterns could indirectly apply to an understanding of cancer origin, according to the authors [29]. All these arguments are in perfect harmony with some assumptions supported by the SMT. However, they are too far from finding a plausible explanation on why one or two starter mutations of cancer, initially planned by the SMT, become now in thousands of changes (mutational landscapes). Thus, it is clear that the SMT initial premises are changing to search for cancer origin between a “regular mutational” background as it will be discussed later. Therefore, it is worth stating that the mutational landscapes should be a “cancer process” consequence and not its origin.

On the other hand, it was previously identified that a significant percentage of human tumors recapitulates the early gene expression of embryogenesis. However, the genes expressed in cancers should have low tissue specificity, distinguishing only three groups of them by expression patterns of different genes during embryogenesis [19]. One group has an early developmental phenotype and expresses specific genes of stem cells. From the embryogenesis viewpoint, the group is very homogenous. According to Naxerova's vision, it is even more surprising as it contains cancer types with complex karyotypes and leads to thinking of a “chaotic” gene expression. A second more heterogeneous group tends to be more similar to the later development and is characterized by an inflammatory gene pattern and, finally, the third group of small cancers presents a transition phenotype between these two extremes and exhibits both characteristics.

Anyhow, the results presented lead to a better understanding of the human disease when there is a macrobiological approach of transcriptome data shifting the focus of individual genes to the biology of embryonic processes. Thus, the transcriptome seems to be useful to establish robust molecular correlations between apparent and unrelated phenotypes of cancer. However, a real macrobiological approach of transcriptome data must take into account obligatorily some aspects of the embryological processes involved, and also, some differences among the animals used in the comparison. For obvious reasons and based on the impossibility of having a human embryo model to study cancer, the mouse became a primary model organism. Nevertheless, the authors will develop some comments/criticisms for those who want to establish a comparison between patterns of gene expression during the mouse embryogenesis and human origin cancer [19].

**Figure 1:**
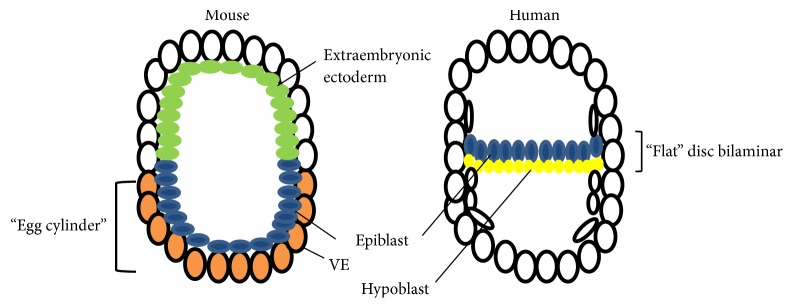
Differences between mouse and human embryos. Mouse embryo has the shape of an egg cylinder (showing the apposition the epiblast and visceral endoderm tissues) and human embryo has the shape of a flat disc with two layers of cells known as epiblast and hypoblast. VE: visceral endoderm. Adapted from Irie et al., [39].

First, it is necessary to consider the significant differences in embryogenesis (Figure 1 adapted from Irie et al. [39]). The human embryo, in the gastrulation stage, has the shape of a flat disc with two layers of cells (bilaminar disk) known as epiblast and hypoblast (Figure 1) [40]. At the beginning of the third week, the primitive streak appears and gastrulation begins (day 14 or 15 postfertilization), and the trilaminar disk will appear as derived exclusively from the epiblast [32]. Nonetheless, in rodents, the embryonic disc has a complex shape called “egg cylinder” (Figure 1), which forms between the fourth and seventh day of pregnancy in vivo [41]. The mouse embryo epiblast organizes initially from a ball of cells and becomes an epithelial layer in the form of a cup (cup-shaped) surrounded by visceral endoderm (equivalent to the humans' hypoblast). Gastrulation begins with the formation of the primitive streak (E6.0 and E6.5) in the epiblast (future embryonic ectoderm) that will give rise to all fetal structures. Such structure contains the precursors of definitive endoderm; however, some visceral endoderm descendants remain in the prospective foregut and hindgut [42]. Furthermore, the visceral endoderm interacts with a second thick-walled and cup-shaped tissue: the extraembryonic ectoderm (ExE) occupies an opposite position to the epiblast (Figure 1), and, apparently, there is no equivalent structure in humans (Figure 1). Thus, the visceral endoderm will also contribute to the formation of pulmonary tissue studied by Naxerova et al., and, therefore, gastrulation in mice occurs in a spatial and temporal configuration that mixes different tissues and interactions when compared to the humans (Figure 1).

The second aspect that can also derive from embryogenesis is that the genetic and genomic differences are recently confirmed between mice and humans. The two species diverged substantially in the gene sequence level. Some research groups had already revealed different patterns of the binding to DNA for a limited number of transcription factors, suggesting significant differences in cellular functions and regulatory mechanisms [43, 44]. Data confirmed by a recent study of the Mouse ENCODE Consortium found that the gene expression and its underlying regulatory programs differed substantially between humans and mice strains. The control programs divergence between mice and humans manifests not only in the gain or loss of cis-regulatory sequences in the mouse genome, but also in the lack of regulatory activities conservation in different tissues and cells types [45] and, also, in the exaptation of regulatory sequences for other distinct functions [46]. The most divergent genes are involved in the extracellular matrix, cell adhesion, receptors to intracellular signaling, immune responses, and other processes related to the cell plasma membrane [45].

Then, when comparisons between humans and mice are established, they should take into account the vast genetic, genomic, and embryonic differences. Thus, since we are discussing the topic below, we agreed that there will be three cancer groups (established during the embryogenesis). To define the three groups, the authors do not use reasons sustained in comparisons between mice and humans but the cell movements during gastrulation leading to a natural result of the process of cellular differentiation and the establishment of three distinct lineages of stem cells, during the embryogenesis period.

## 3. Cancer as an Embryological Phenomenon

Currently, the developmental biology deals with cells growth control, their differentiation, morphogenesis, and organogenesis, a process that gives rise to the formation of tissues and organs. Then, cell differentiation is a fundamental issue of developmental biology [32, 47] as well as cancer. It is naturally accepted that tumorigenesis derives as a disruption result of the normal process of cell differentiation [48–50] that should be controlled by regulatory networks of developmental genes. Thus, these developmental genes could be important determinants in cell differentiation, with a vital role in tumor initiation and progression [51, 52].

The historical roots of our understanding of the intimate relationship between tumorigenesis and development processes date back to 1859 when Rudolf Virchow called neoplasia as “pathological new-formations,” and they appeared “by the same law that regulated the embryonic development.” [53]. In 1892, the French biologists Lobstein and Recamier also speculated with the notion of tumors embryonic origin [54]. From the authors' point of view, between the years 1967 and 1974, Pierce proposed a theory that links cancer to developmental biology [50, 55], noting that tumorigenesis could have its cause or origin closely related to developmental biology and cell differentiation: “…the mechanism of normal cell differentiation and stabilization apply to the pathological differentiation and stabilization of cells when they become cancerous and strongly suggest that concepts of developmental biology will provides new approaches to therapy for cancer” [55], and he even speculated on cancer stem cells.

*Evidence has been presented to support the concept that malignant tumors are postembryonic differentiations superimposed upon the process of tissue maintenance and renewal. Malignant stem cells are derived from normal stem cells. They have a capacity for proliferation and differentiation that operates at a different level of control compared with the normal. Even so, malignant stem cells are responsive to environmental control, suggesting that it may be possible to direct their differentiation or at least to control their ability to replicate. A tumor is a caricature of normal tissue and appears undifferentiated because of the preponderance of undifferentiated proliferating stem cells in relationship to the number of cells that have differentiated and become benign* [50].

Moreover, Pierce did not believe that mutations or viral inserts were the cause of cancer. For him, the phenotypic traits of malignant cells seemed to be encoded in the genome of normal cells, favoring the idea that the production of a neoplasm is probably similar to the production of any normal tissue [56]. The mechanism of tissue genesis involves cell division, differentiation, and organization. In other words, carcinogenesis could be an epigenetic event similar to the postembryonic differentiation. Therefore, some experimental results indicated that the formation of cancer cells could be the result of repressed genes reactivation, during normal embryonic development [57–59].

A body of evidence confirmed the correlation between embryo development and tumorigenesis with the advance of molecular biology, tumor immunology, developmental biology, and experimental embryology. Similarities between cancer and development are evident in many microscopic observation levels. Despite the fact that there are significant differences between normal and cancerous cells such as more metabolic autonomy and the activity increase of the phosphatidylinositol 3-kinase (PI3K) system (through a variety of mechanisms) in cancer [13], cancerous tissues appear as undifferentiated masses, and some types of tumors even present an embryonic tissue organization. The increased mobility of malignant cells directly correlates with invasiveness, thus producing a metastasis with potential to spread to distant organs (poor prognosis of cancer). It is also one of the main features of the migratory behavior of embryonic cells during gastrulation and expressed by the invasive capacity in a faraway location that will help to form the embryonic endoderm. At the molecular level, common characteristics between certain malignant tumors and developing tissues about the transcription factors activity [60], regulation of chromatin structure [61, 62], and signaling [63] have already been widely documented. On the other hand, several papers have suggested that cancer transcriptome represents a “development signature,” that is, a set of genes activated simultaneously during the embryogenesis [14–16].

Nowadays, we also know that stem cells in adult humans are really the custodians or remnants of the embryo's “modus operandi” to build and leave possibilities of rebuilding, at different times, in organs and tissues levels. Thus, it is possible to find transcriptional regulatory mechanisms in stem cells for the simultaneous control of cell growth, migration, and differentiation. These regulatory mechanisms could be extremely crucial for organs maintenance and regeneration, mainly, because such mechanisms might be reattached again when they are necessary for the adult context. Also, it is not surprising, in this context, that, in the so-called “cancer stem cells,” we may find today one of the main inspirations about cancer origin [4, 50, 64–71]. The authors speculate that within these cells the process that triggers cancer disease may begin, and, probably, it is bound to an anomalous regulation of cell growth and cell differentiation induced by environmental stimuli as we are going to see later.

Then, we suggest that cancer types might be associated with three different groups established coherently during the embryo gastrulation. Therefore, we propose cancers of ectodermal, mesodermal, and endodermal origins. The conceptual assumptions of our proposal are the following: (1) the proliferation is by default state of cells; however, it happens associated with a specific cellular destination established in the gastrulation. (2) Cells establish tissues during gastrulation and create different control mechanisms of their organization and future reorganization (remodeling process). Thus, the healing of skin wounds [72] and remodeling of endodermal lung epithelium in cases of cystic fibrosis, desquamative interstitial pneumonia [73, 74], or breast remodeling during puberty or pregnancy [75] are possible by the activation of these control mechanisms of tissue reorganization. Therefore, in the context of “cancer disease,” carcinogens break these control mechanisms or “controlling forces” [76, 77] and create an unnecessary process in an improper context. Such idea is not new in the biological sciences, and a classification of this type was proposed by Whitney, in 1901, identifying cancerous tissues as epiblastic, mesoblastic, and hypoblastic [78].

**Figure 2:**
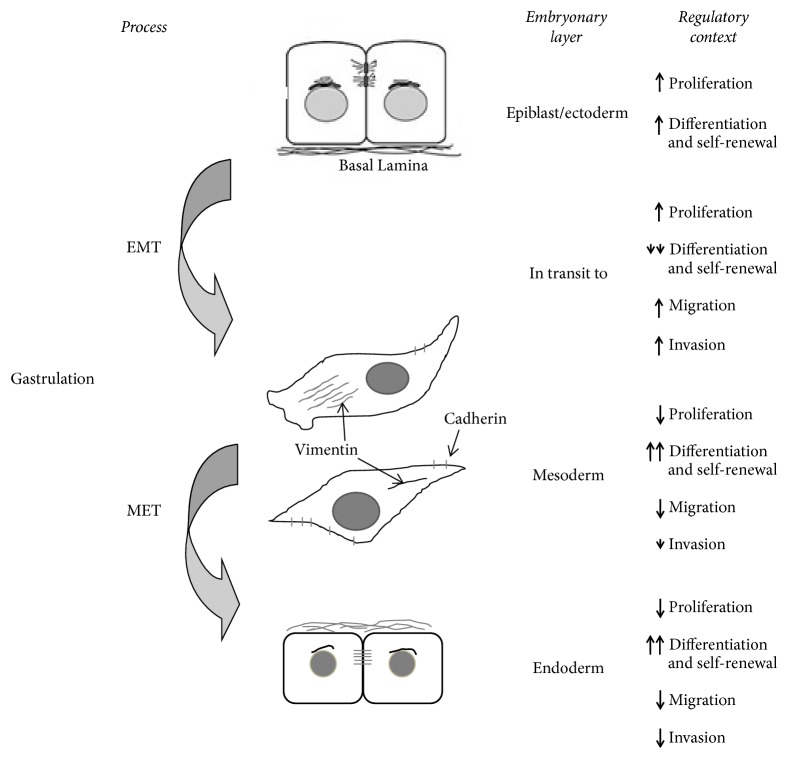
Differences established during gastrulation through the EMT and MET processes. The EMT and MET processes involve a specific transcriptional regulation and they are responsible for simultaneously orchestrating processes of proliferation, migration, cell differentiation, and invasion. However, these programs are different depending on the embryonic layer of origin. The arrowhead or arrow indicates the “intensity” of the regulation.

**Figure 3:**
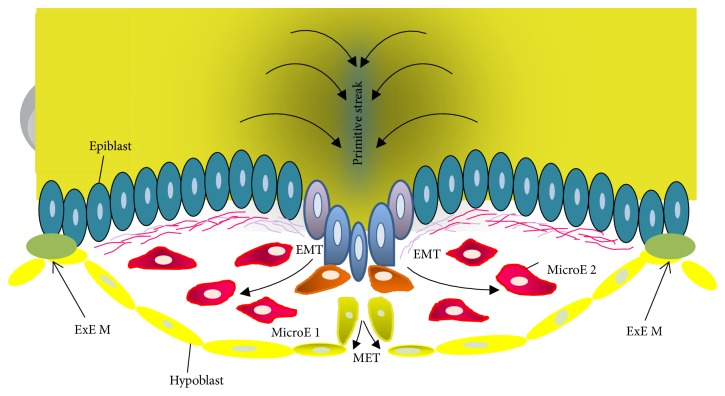
Human gastrulation. During gastrulation, epiblast cells at the primitive streak undergo Epithelial-Mesenchymal Transition (EMT) as a result of signals produced by the Spemann organizer or internal determinants. The migratory mesenchymal cells are interacting with the embryonic context in a different way: microenvironment 1 (MicroE 1), only with the hypoblast, exactly, in the middle region of the embryo. Microenvironment 2 (MicroE 2), simultaneously with the hypoblast and the extraembryonic mesoderm (EXE M) in lateral ends of the embryonic disc. Adapted from Micalizzi et al., [79].

Then, a fundamental aspect of our hypothesis on cancer origin is the Epithelial-Mesenchymal Transition (EMT) and Mesenchymal-Epithelial Transition (MET) that occur during gastrulation [79, 80]. EMT and MET processes involve a specific transcriptional regulation (EMT and MET programs) [81], and they are responsible for simultaneously orchestrating processes of proliferation, migration, cell differentiation, and invasion (beginnings of the natural ability of metastasis of human cells). However, these programs are different depending on the embryonic layer of origin by our hypothesis (Figure 2). All epiblast cells have the potential to perform the EMT before starting the gastrulation. But, only a few epiblast cells will ingress through the primitive streak and acquire mesenchymal characteristics (Figure 3). Therefore, these mesenchymal-like cells give origin to the mesoderm (dermis, muscles (smooth and striated), cartilage and bone, and other tissues) [82] and the endoderm (intestine epithelium and associated organs) [83]. On the other hand, the remaining epiblast layer, formed by those cells that did not perform EMT, will form the embryonic ectoderm to differentiate the epidermis [84] and neural plate [85], in the future (Figure 3). In these ectodermal cells, the EMT program must be actively suppressed to ensure the proper differentiation of epidermis and neural tube (Figure 2). In Figure 3, it is possible to observe that migratory mesenchymal cells are also proliferating, but, especially, they are interacting with the embryonic context in a different way. For simplicity reasons, we emphasize that in the primitive streak region, where the cells are delaminating, two different types of interactions are possible: the microenvironment, 1 only with the hypoblast, exactly, in the middle region of the embryo (Figure 3) or the microenvironment 2, simultaneously with the hypoblast and the extraembryonic mesoderm in lateral ends of the embryonic disc (Figure 3). We think that mesenchymal cells interpret these specific microenvironments (1 and 2) in different ways, depending on their developmental history after the primitive streak delamination. Then, the result will be different responses in mesenchymal cells, in microenvironments 1 and 2. Therefore, they will contribute to producing a change in the epigenetic state [35] and create different patterns of transcriptional regulation [86, 87] that will allow the efficient differentiation of mesodermal and endodermal lineages.

Consistent with the establishment of these lineages differences, after delamination by the primitive streak, “preendodermal” mesenchymal cells will acquire some additional features that will allow triggering the MET. In other words, they gain an invasiveness displayed only by cells “en route” to substitute the original hypoblast and form the new and definitive endodermal epithelium (Figure 3) [88]. It is noteworthy that the transcriptional regulation should be initially similar to mesodermal and endodermal cells because similar processes such as differentiation, proliferation, and migration are being regulated [89]. However, some significant molecular differences are established by involving, mainly, Smad4 and Mixl1 [90, 91] before starting the MET in both lineages. Then, it seems to be a causal relationship between the control mechanisms of differentiation, remodeling capacity of the basement membrane (BM), and migration [90]. That is confirmed because the functional loss of Smad4 produced a defect in the endodermal commitment that can be directly associated with the inability to break down and remodel the BM by downregulation of the expression of matrix metalloproteinase-14 (MMP14) enzyme and matrix metalloproteinase-9 (MMP9) enzyme. Also, in the Mixl1-null mouse embryos can be confirmed the independence of migratory movements of embryonic endoderm and mesoderm [91], suggesting that the establishment of the two lineages could be originated before the hypoblast displacement [88, 90, 91]. These differences in the BM remodeling program between mesodermal and endodermal lineages constitute one of the critical aspects of our theoretical framework. Therefore, in our model, the endodermal cells have an additional capacity of remodeling the BM that will produce significant invasiveness difference in cancer of endodermal origin by triggering a worse prognosis for patients with that type of disease. Finally, we believe that gastrulation could be the embryological process that generates different epigenetic signatures in all three germ layers (ectoderm, mesoderm, and endoderm), and they manifest immediately in several different capacities for remodeling the BM as a response to signals and microenvironmental conditions within the embryo. In the future, such different capacities will manifest in various potentialities to develop cancer in the human population.

Therefore, it is possible to predict that cancers of endodermal and mesodermal origin are always the worst because their establishment is being reactivated by a lineage-specific transcriptional mechanism (responsible for the regulation of differentiation, migration, and invasion) present in the stem cells of the tissues formed in these embryonic layers. We also believe that the endodermal cells possess an additional transcriptional mechanism that finely modulates the transition between the two processes (EMT and MET), and, therefore, the prognosis will be worse in cells of endodermal origin. Mesenchymal cells only perform the EMT; however, it is worth mentioning that according to the current epidemiological situation, we must be cautious in saying that some mesenchymal cells will make the MET. The ectodermal cells do not require basal lamina breakage to produce their differentiation and tissue organization. Then, cancers will always be less aggressive and with lower mortality as, for example, the nonmelanoma skin cancers (basal cell carcinoma) [10, 92], however, with a few exceptions as we shall see.

When there is an analysis of the list of the most common types of cancer, including those more often diagnosed in Brazil and excluding the nonmelanoma skin cancer, it is possible to observe that, among the top ten cancers with the highest incidence in men, eight of them are of endodermal origin (EMT and MET) (Prostate, 22,8; Trachea, bronchus, and lung 5,4%; Colon and Rectal 5,0%; Stomach 4,3%; Oral Cavity 3,7%; Esophagus 2,6%; Bladder 2,2%; and Larynx 2,3%), one derived from two origins (mesodermal/endodermal or their interaction) (Leukemia 1,7%) and one of ectodermal origin, representing only 1.6% of all cases registered in 2014 [10]. For women, among the top 10 cancers, the highest incidence four are of endodermal origin (Colon and Rectal 6,4%; Trachea, bronchus, and lung 4,0%; Thyroid 2,9%; and Stomach 2,7%), three are of mesodermal origin (Cervical 5,7%; Uterine body 2,2%; and Ovary 2,1%), two are derived from mesodermal-endodermal interaction (Non-Hodgkin lymphoma 1,8% and Leukemia 1.6%), and one is from ectodermal-mesodermal interaction (Female Breast 20,8%;) [10].

The panorama is very similar in the United States with some regional differences expected, considering environmental and lifestyle influences. Thus, among the ten most commonly diagnosed cancers in men (excluding basal cell and squamous cell skin cancers), six are of endodermal origin (EMT and MET) (Prostate, 26%; Trachea, bronchus, and lung 14%; Colon and Rectal 8,0%; Bladder 7%; Oral Cavity 4%; and liver and intrahepatic bile duct 3%), one is of mesodermal origin (EMT) (Kidney 5%), two are derived from two origins (endodermal-mesodermal interaction) (Non-Hodgkin lymphoma 5%; Leukemia 4%), and one is of ectodermal origin (melanoma), representing 5% of all cases registered in 2015 [92]. For women, among the ten most frequently diagnosed cancers four are of endodermal origin (Colon and Rectal 8%; Trachea, bronchus, and lung 13%; Thyroid 6%; and pancreas 3%), two are of mesodermal origin (Uterine body 7% and Kidney 3%), two are derived from two origins or mesodermal-endodermal interaction (Non-Hodgkin lymphoma 4% and Leukemia 3%), one is derived from ectodermal-mesodermal interaction (Female Breast 29%), and one is of ectodermal origin (melanoma 4%) [92]. Finally, comparing Brazil to the Unites States, stomach cancer is an important health problem among women and men in Brazil, and melanoma, pancreas, kidney, and liver cancers are very incident among women and men in the United States.

Now, considering the leading causes of death from cancer in Brazil and the United States, it becomes even clearer the advantage offered by the separation in three groups of cancer based on embryonic origin (and their different transcriptional regulation profiles in the EMT/MET). In Brazil, according to 2010 data and considering the total number of cancer deaths, 64% of all male cancer deaths and 33% of all female cancer deaths were due to endodermal origin cancer [93]. In 2015, the estimation is that around 312,150 men will die from cancer in the United States, and 65% of them will die from endodermal origin cancer, thereby representing 202,897 American men dead. Among women, endodermal origin cancer will represent 45% of all deaths [92]. Thus, the aggressiveness and invasive capability could be present in endodermal cells, and the ability to reactivate their potentiality in a particular organ or tissue can occur as a result of a whole range of environmental and lifestyle influences from different countries such as Brazil and the United States.

The mixed feature of some cancers such as leukemic and breast ones was supported in the last years. The initial set of hematopoietic stem cells (HSCs) is initially formed during embryogenesis in mesoderm and could involve various anatomical sites (yolk sac, aorta-gonad-mesonephros region, placenta, and fetal liver), after which the HSCs finally colonize the bone marrow at birth [94, 95]. Nevertheless, the specification of hematopoietic destination in the yolk sac depends on the visceral endoderm and their specific signs such as Ihh (Indian hedgehog) [96, 97] and BMP4 (bone morphogenetic protein 4) [98], demonstrating the importance of an appropriate mixed microenvironment for hematopoietic commitment. Also, the liver (tissue of endodermal origin) [99] supports the hematopoiesis during fetal life through mixed cells of mesodermal-endodermal origin derived from the hepatic stroma [100]. On the other hand, the human breast consists of parenchyma and stroma, with ectodermal-mesodermal origin, respectively [101]. We also know that the breast cancer invasive capacity involves both epithelial and stromal changes [102–104], and such alterations in different tissues could play a significant role in the establishment of abnormal tumor microenvironment. Thereby, it might contribute to the tumor progression [102, 103].

Finally, it is also necessary to describe cancers of ectodermal origin. Due to their importance in the context of our theoretical model, we are going to describe some of the embryological fundamentals of ectoderm differentiation. Spemann's experiments, in 1924, suggested that the dorsal blastopore lip represents a differentiation center from which the determination process gradually expands toward the nondetermined ectoderm, in the gastrula stage [47]. In the current view, neural induction divides the ectoderm into three destinations: neural crest, neural plate, and epidermis [105].

The neural crest cells, after differentiation, form many derivatives prone to malignant transformation, including melanocytes that can form melanomas and glial cells that may develop Schwannomas and gliomas. In this context, melanoma is a type of invasive cancer [70, 106] because the neural crest cells perform the EMT from the neural tube [107–109]. Therefore, they acquire, in a different time other than the suggested by our model, the capacity of simultaneous regulation of differentiation (multipotency maintenance), proliferation, and migration [110, 111]. It is worth pointing out that neural crests are a population induced at the edge of the neural plate [112] and, in the case of melanoma, these are ectodermal cells very similar to mesodermal ones of the model discussed in the present paper [105]. Concerning the cancer process issues, it is significant to know better the molecular mechanisms responsible for melanocytes induction [40, 113]. Furthermore, features and migration routes [114] could explain the differences between melanomas and gliomas.

Then, for the comprehension of human cancers of the central nervous system and epidermis (it may seem contradictory because they are cells that do not perform the EMT), it is necessary to take into account two fundamental aspects: (1) conservation/maintenance of the EMT in a suppressed state in these tissues and (2) evolution of the stem cell concept. Regarding the first aspect, most neurons are produced in distant locations from their final ones. Therefore, migration is a fundamental and common property of newborn neurons. Recent studies claim that the early neuronal migration can be regarded as the EMT-like phenomenon, in which cells lose epithelial characteristics and acquire mesenchymal features [115], and, also, the migration control in these cells could be intimately related to cell differentiation [116]. On the other hand, it is significant to consider that the spatial organization of the neocortex also requires the migration of various types of neuronal cells produced in the ventricular zone and sent to appropriate layers in the cortical plate, and the EMT concept could also have an impact on the outer radial glia [115]. In the case of epidermal epithelial cells and female mammary gland, it is known that they suppress the EMT actively to ensure differentiation from the normal epithelium [117, 118]. Thus, our theoretical model is compatible with the establishment of a mechanism of a different tissue reorganization/organization in the ectodermal layer that could handle some types of invasive tumors generated by cells with such embryonic origin.

The second important aspect to consider is that the stem cells biology may be more complicated than originally planned. The discovery that stem cells can reside first in a tissue, in adults, and by traveling through the bloodstream may contribute to the formation of another tissue suggests a degree of plasticity not previously recognized in the stem cells function [119]. Then, it seems that the stem cell destination could change all the time as a natural property of these cells, thus resulting in a new form of interpreting their involvement in the physiological repair of tissue damage throughout life [120, 121]. A “plastic property” of these cells was predicted at the beginning of experimental biology as we shall see. However, some embryological inductive studies by Holtfreter [122], Nieuwkoop [123], Smith [124], Slack [125], and Gurdon [126] seem to have left the false impression that specific stem cells of an organ are restricted to produce differentiated cells types of the tissues in which they reside (developmental restriction). The papers mentioned before are general studies to understand the signals involved in cell differentiation during embryogenesis, and they did not have the purpose of researching stem cells. In other words, it makes no sense to assign for these results the variables not established by the authors as, for example, the stem cells appear to have irreversibly lost the capacity to generate other cell types in the body. Embryological classical studies using pieces of isolated tissue or in vivo tissue verified in a tissue level or at an organismic level that tissues never lost the integrity and never turned into other tissues after differentiation.

Therefore, developmental restriction apparently originates from a misinterpretation of biological processes that take place on a cellular level from experiments on a tissue level. Such consideration is so significant that the concept of developmental restriction of differentiation does not have any support in the origin of experimental embryology, and Spemann wrote, in 1924, regarding the “differentiation center” in the dorsal lip of the blastopore that

*the designation “organizer” (rather than, perhaps, “determiner”) is supposed to express the idea that the effect emanating from these preferential regions is not determinative in a definite restricted direction, but that it possesses all those enigmatic peculiarities which are known to us only from living organism* [47].

Then, in 1924, Spemann seemed to predict the enigmatic plastic peculiarity of stem cells [120, 121] that may have important implications in the way of explaining cancers of the nervous system and skin. Thus, for example, bone marrow cells of mesodermal/endodermal origin [100] infiltrate the brain and differentiate into cells that express neuronal specific antigens [127, 128]. In the epidermis the presence of pluripotent stem cells with mesenchymal characteristics confirmed by the expression of specific molecular markers of that tissue and also by morphological characteristics was found. So far there is no explanation for its origin [129].

Therefore, our model predicts that epidermal cancers should not be invasive and could have a very low mortality rate for their embryonic origin. That is observed in nonmelanoma cancers, particularly, basal cell cancers (basal cell carcinoma) that are the most prevalent in men and women and the least aggressive, with a good prognosis [10, 92]. However, epidermoid cancers (squamous cell carcinoma) are more invasive, and we speculate that could be caused by stem cells secondarily incorporated into the skin [129], during the life of the human being. Something similar might also happen in the central nervous system and could explain that, in the world, this type of cancer represents approximately 1.9% of all malignant neoplasms [10, 92].

## 4. The Embryological Model of Cancer: Recontextualizing Pierce's Ideas

**Figure 4:**
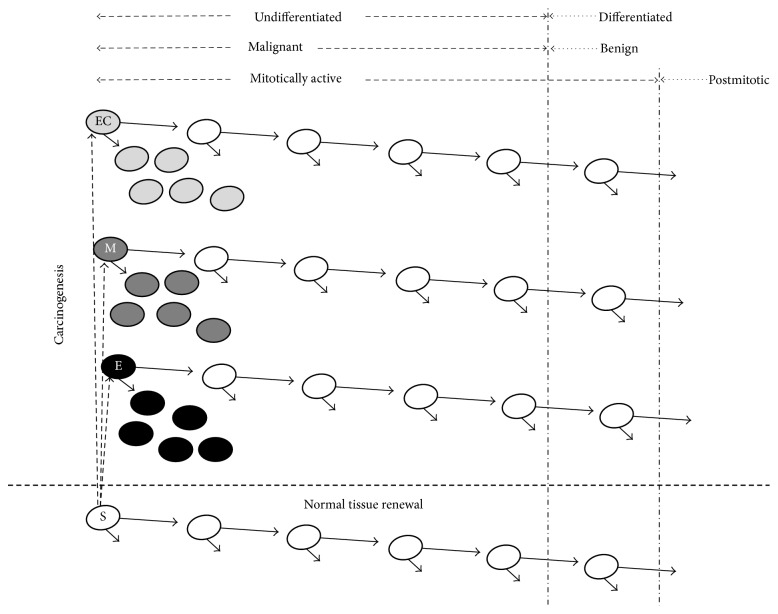
Embryonic model of cancer. The normal cell lineage of tissue renewal is at the bottom. The three different tumors are at the top. The different types of cancer correspond to their different embryonic origins. S: normal stem cell; E: tumor of the endodermic origin; M: tumor of the mesodermic origin; and EC: tumor of the ectodermic origin. Model adapted from the original idea of Pierce, 1983.

Cancer disease could be an altered representation of the normal process of tissue renewal, following the conceptual definition of Pierce (Figure 4). The usual process of tissue renewal is essential for the viability of complex organisms, and, also, it allows the substitution of a precise number of cells that become senescent and with different characteristics if they came from different embryonic layers. Furthermore, the malignant stem cell derives from the regular stem cell. It resembles the normal one, and its mechanism of growth control could allow that cancerous cells to divide faster than the normal stem cells, resulting in the undifferentiated phenotypic aspect of the tumor (Figure 4). The model also foresees that malignant tumors contain cancerous cells from the beginning, and these cells could be in a number considered as a subthreshold to express their phenotype, and, thus, tumors behave in a benign way, at least initially [56]. Consequently, more than one cell type can be involved in oncogenesis, and tumors with multiclonal origin could become monoclonal ones over time. The latter should be approached carefully, with an emphasis in the exact moment of tumor initiation and without considering three aspects: (1) the long history of its consolidation, (2) chromosome instabilities resulting from that history, and (3) the transdifferentiation process that redirects cells destination in the tumor center, for example, to produce endothelial cells [130], which are the first ones to differentiate during embryogenesis.

**Figure 5:**
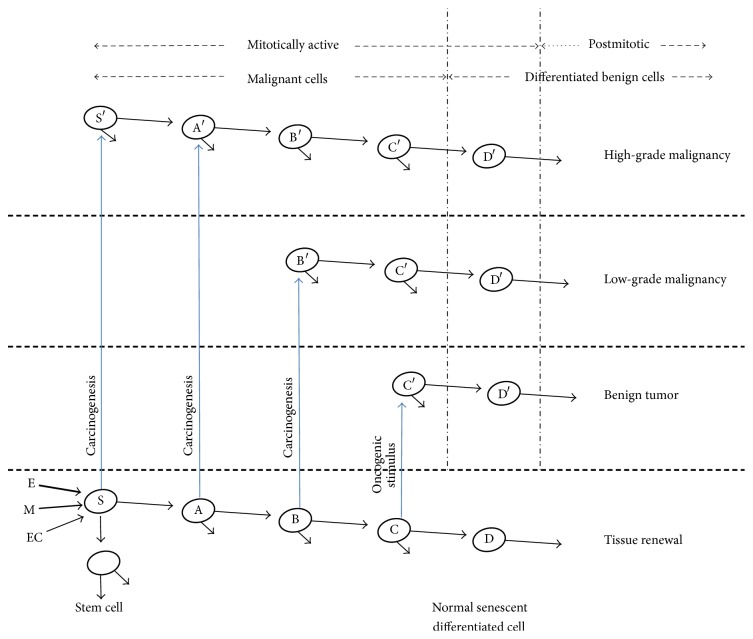
Differentiation and neoplasia. The normal cell lineage is at the bottom. Carcinogenesis may involve S, A, B, or C cells individually, giving origin to their corresponding malignant cell types. Their potentials for differentiation are indicated by the arrows. The cancers of endodermal and mesodermal origin are always the worst (thick arrow) in comparison with the ectodermal cancers (thin arrow) with a few exceptions as outlined in the article. EC: ectodermal origin; M: Mesodermal origin; and E: endodermal origin. Adapted from Pierce, 1983.

In other words, such concept could be helpful to understand the origin of benign and malignant tumors because it predicts that these two types of tumors arise from cells transformation at different stages of differentiation (Figure 5), which is entirely possible to happen in the cancer formation context in an adult tissue. Thus, the benign cells could form by the transformation of cells closer to the final stage of differentiation and cancerous cells closer to the stem cell stage (Figure 5). That fits perfectly into our model of three embryonic origins of cancer and could be valid in the independent context of ectodermal, mesodermal, and endodermal tumors, with the expected differences to the intrinsic transcriptional regulatory control of each one of these embryonic layers. By the present proposal, invasiveness and aggressiveness capacities depend on the embryonic layer of origin. We claim to only expand the conceptual model presented by Pierce in 1974 and readjust it to a new form of embryological interpretation (Figure 4) [50].

Also, it is possible to observe some significant considerations about the differentiation concept as a mechanism of carcinogenesis by rereading Pierce's work. At that time, Pierce acknowledged that it was an idea that “has not been disproved” and the possible dedifferentiation of C′ in S′ (Figure 5) from his point of view “what has been interpreted as dedifferentiation is, in reality, an abortive attempt of differentiation.” However, we know today that cells already differentiated can spontaneously acquire stem cells characteristics as demonstrated with human mammary epithelial cells [131] and mouse spermatogonia [132] and, surprisingly, the neoplastic transformation improves that simple phenotype conversion. Moreover, some experimental results from our laboratory show that the functional block of Kaiso and p120ctn in K562 cells, established as a model of chronic myeloid leukemia in blast crisis, could help in maintaining an undifferentiated state of these transfected cells. One of the interesting aspects of Kaiso's functional block and p120ctn with siRNA in K562 is the significant decrease in the CD33 expression on the cell surface since the commitment of hematopoietic myeloid progenitors is characterized by the loss of CD34 and gain of CD33, in the plasma membrane. It was consistent with a more undifferentiated appearance of the K562 cell, after Kaiso's block and p120ctn, the significant decrease in the expression of C/EBP*α* and a 70% increase in the expression of C-MyB. On the other hand, Kaiso's functional block and p120ctn produced an improvement in the cell proliferation capacity, and, then, the phenotypic aspect of the plasma membrane and increased survival of cancer cells K562 transfected with siRNA recall more cancerous stem cell [133].

At the same time of Pierce's work consolidation, there was a discussion about the retrodifferentiation concept, which appeared as an adaptive process involved in maintaining the cell integrity against harmful agents of varied etiology, including physical and chemical aspects and also viruses. Even preserving all the information encoded in the genome, cells undergoing retrodifferentiation could lose morphological and functional complexity by a process of “self-deletion of cytoplasmic structures” and a transition to a “more juvenile” pattern of gene expression [134]. Nowadays, the concept can be translated as cellular plasticity or dedifferentiation and represents a profound divergence from the unidirectional and hierarchical model accepted for cell differentiation. The connection between that concept and cancer was described poetically by Uriel, who wrote in 1976 that “cancer may thus be regarded as the myth of Faust on the cellular level, the cells' chimeric dream of rejuvenation and immortality, which, in the end, often turns into a fatal nightmare.” It could have a significant meaning in the therapeutic implications of cancer because there would be a smooth and intricate mixture of two models, the model of stem cells and differentiation and the retrodifferentiation one, a mixed model with a lot more sense in the current epigenomics conjuncture of the XXI century [135–138].

Finally, in an attempt to understand the molecular mechanisms of the plasticity found in cancer cells, Dr. Weinberg suggests the “contextual signals” of the tumor microenvironment in vivo are capable of causing the Epithelial-Mesenchymal Transition (EMT). It sets up an implicit recognition of the close link between the passage through an EMT and induction of multipotent populations of stem cells, during gastrulation [135, 139]. Thus, the integration of control mechanisms of cell differentiation and/or cell plasticity with embryogenesis could first understand cancer as a result of reactivation of cells standard processes without the intermediation of mutations, and, second, they might represent not only plasticity but also differentiation in an attempt to explain the cancer origin.

## 5. Concluding Remarks

### 5.1. The Weakness of Our Experimental Models

It is worth stating that one of the most significant limitations to consolidate an embryonic cancer study is the lack of a human experimental model influenced by distinct ethical aspects that focused or restricted studies with human embryos [140–143]. On the other hand, research groups working with hESC cannot consider the inner cell mass of blastocysts as embryos but only as a group of cells in which the priority was always the creation of cell lines and the impact on biomedical research [142, 144, 145], and also with a poor attitude of understanding what they truly are or represent and their specific role in the embryonic context. It is a concept mistake with grave consequences because we are losing the opportunity to meet some determinants of internal order and internal forces that might contribute to the understanding of significant aspects of cell differentiation, human morphogenesis, and cancer as we shall discuss in a future article. It is worth noting that perhaps the rebellion presented in chromosomal instability of hESC in culture is an invitation to consider these cells a whole inseparable and mutually dependent [146–150]. Some examples in need to look at are the set of hESC that comes up with papers on mouse stem cells (mESC). The mESC, cultivated with serum but without supplementation of growth factors, differentiate spontaneously in hepatocytes with similar and comparable functional properties to those obtained when differentiation was guided by growth factors [151]. In relation to the progress in mouse embryogenesis, it is worth highlighting Dr. Zernicka-Goetz's work that shows and illuminates the internal determinants in embryo formation forcefully (about the prospective morphogenetic potency described by Hans Driesch in 1892 [152]) and, also, the way to better use human embryos for scientific research to focus on cell differentiation and cancer [153–159].

Finally, it is worth mentioning, at the end of our reflection, the words of Joseph Needham who showed, in 1936, the need to study the embryo as a set of relations, and he also suggested that in these internal relations, we shall find cancer control.

*Processes by which different parts of an organizer induce the formation of different organs are called “individuating actions,” and the organizer has a certain relation to the plan of the whole body which we may speak of as existence in an “individuation field”. The main characteristic of an individuation field is that all tissue lying within it tends to be built up into one complete embryo, and in any one part of the field all tissue tends to be built up into the organ corresponding to that part. These parts of the field are sometimes called are-as or districts (e.g. the fore-limb area) and they are, as far as we can see, the controlling forces from which the cancerous growth has escaped*. [77].

### 5.2. The Weakness in the Consolidation of Theoretical Foundations

The authors agree with the statements that most scientists “make no efforts to find theoretical underpinnings in their daily bench experience” and that “the overwhelming majority of biologists either tacitly or explicitly adopt the ontological position that what actually exists is matter. Within this materialist stance, the dominant epistemology is reductionism” [160]. And both are failures of our day-to-day work as scientists, and they end up rebounding when we want to build a plausible explanation of the facts. When reductionism is big the possibility to reconstruct the whole in a coherent manner is small. When the number of variables is large, the uncertainty of interpretations regarding the complex nature of a phenomenon such as cancer is also significant, and it is reinforced by the lack of knowledge on the disease origin, and, thus, it achieves the “possible limit” in the epistemological reductionism. Therefore, it is time to change the science construction strategies. Consistent with these arguments, scientists do not make efforts to recapitulate all the facts and harness the ideas in the large lines of evidence already present in the literature as background. Moreover, we usually discard the construction of explanatory models to understand what we are studying and, more importantly, to understand why we are studying it.

Then, “the dilemma of cancer research is exemplified by the increasing obscurity of much of the writing, by the extraordinary remoteness, range, and intricacy of the lists of papers presented at cancer meetings and by their failure to illuminate the scene. Information accumulates space while understanding lags behind” [161]. Therefore, half a century later, it seems that little progress has been developed and the prevailing theory of somatic mutation was not yet rigorously tested, and several lines of evidence raise questions that are not addressed by that theory [162]. For example, mutations also occur in normal cells and thousands of somatic single nucleotide variants (SNVs) were identified in the cerebral cortex neurons of three normal individuals, which seemed to represent the cell state with somatic mutations. It was a permanent and durable record of the neuron natural history from the embryonic development to its postmitotic functional state [163]. These mutations appear to be part of the cell's normal and physiological context. Thus, it could be important to answer the following question: how do mutations integrate to the physiological context of a cell? In the cancer context, it is particularly evident in the study of acute myeloid leukemia (AML), in which there is a lack of reliable theoretical models that integrate mutations to the normal cellular context, “our data suggest that most of the somatic events in AML genomes appear to be random, preexisting, background mutations in the hematopoietic cell that acquired the key initiating mutation” [164]. Therefore, cancer could not be originated by one or two mutations, but because one or two mutations were selected from hundreds or even thousands of them. Thus, a small fraction of the total mutations in each AML genome should be relevant to the pathogenesis, and we do not understand what to do with the vast majority of that mutational background. We need to highlight two remarkable aspects of this study. First, the huge distance between results and foundations established by the theory of somatic mutation [165]. Second, and not related to the AML, is that these results point out mutations as neutral, tacit recognition within cancer studies from Kimura's theory, whose primary tenet is that the vast majority of evolutionary changes at the molecular level are caused by random attachment of alleles selectively neutral through random sampling drift under continuous mutational pressure [166].

Finally, it is worth noting that cancer stem cells hypothesis has been approached very strongly from the experimental point of view, and we consider it as one of the great strengths of current science (very well reviewed about melanoma in Shakhova and Sommer [106]). Nevertheless, the embryological origin of cancer does not have any prominence nor seems to be in the interest of basic and applied research area from the theoretical point of view. Despite the theoretical weaknesses, some efforts were devoted to change the hierarchical level of cancer and put it correctly on a tissue level of organization. In that context, it is significant to emphasize the Tissue Organization Field Theory, an extraordinary theoretical and conceptual base that helps to resize carcinogenesis as a problem of tissue organization similar to organogenesis [160, 162]. The authors' model, in that sense of changes in the hierarchical level, points out that cell differentiation [48, 55], during embryonic morphogenesis, establishes an array of different relations in a tissue context (germ layers), and it influences, for example, the prognosis of cancer disease in case it happens. Seduced by Pierce ideas, we purposely focused on cancer phenomenon and the particular occurrence in stem cells originated during embryogenesis. However, it is worth recognizing that any effect on the stem cells will only make sense if the relations in tissue context, in which they are inserted, become affected. Finally, it is possible to maintain by following the experimental embryology tradition that cancer is a peculiar phenomenon of our embryological origin. Therefore, it is also intrinsic to our lives as human beings, and it happens regardless of any genetic mutation during normal embryogenesis. Thus, cancer disease can be the awakening of a cellular mechanism in an inappropriate context and time. Among the forms of awakening of cellular mechanism, the environmental stimuli seem to manifest through genetic or epigenetic alterations. It is necessary to be sure that it is going to be awakened a direct reflection of our embryological origin, and mutations or epimutations are just instruments to express the different potential that each germ layer possesses. At least, we hope our cancer vision helps to change the experimental models allowing studying the disease without natural distortions and biases that attribute the cancer cause to genetic phenomena. It is by recognizing their embryological origin that studying models of cancer are going to be reassessed, and, thus, the scientific community as a whole is going to produce a profound impact on future cancer therapy.
